# AM-2201 Inhibits Multiple Cytochrome P450 and Uridine 5′-Diphospho-Glucuronosyltransferase Enzyme Activities in Human Liver Microsomes

**DOI:** 10.3390/molecules22030443

**Published:** 2017-03-10

**Authors:** Ju-Hyun Kim, Soon-Sang Kwon, Tae Yeon Kong, Jae Chul Cheong, Hee Seung Kim, Moon Kyo In, Hye Suk Lee

**Affiliations:** 1 Drug Metabolism and Bioanalysis Laboratory, College of Pharmacy, The Catholic University of Korea, 43 Jibong-ro, Wonmi-gu, Bucheon 14662, Korea; jhyunkim@catholic.ac.kr (J.-H.K.); zuzutnseo@naver.com (S.-S.K.); kongtaeyun@naver.com (T.Y.K.); 2 Forensic Chemistry Laboratory, Forensic Science Division, Supreme Prosecutor’s Office, 157 Banpo-daero, Seocho-gu, Seoul 06590, Korea; Saturn-jjc@spo.go.kr (J.C.C.); hskjay@spo.go.kr (H.S.K.); inmk@spo.go.kr (M.K.I.)

**Keywords:** AM-2201, cytochrome P450 inhibition, UDP-glucuronosyltransferase inhibition, human liver microsomes, drug-drug interaction

## Abstract

AM-2201 is a synthetic cannabinoid that acts as a potent agonist at cannabinoid receptors and its abuse has increased. However, there are no reports of the inhibitory effect of AM-2201 on human cytochrome P450 (CYP) or uridine 5′-diphospho-glucuronosyltransferase (UGT) enzymes. We evaluated the inhibitory effect of AM-2201 on the activities of eight major human CYPs (1A2, 2A6, 2B6, 2C8, 2C9, 2C19, 2D6, and 3A4) and six major human UGTs (1A1, 1A3, 1A4, 1A6, 1A9, and 2B7) enzymes in pooled human liver microsomes using liquid chromatography–tandem mass spectrometry to investigate drug interaction potentials of AM-2201. AM-2201 potently inhibited CYP2C9-catalyzed diclofenac 4′-hydroxylation, CYP3A4-catalyzed midazolam 1′-hydroxylation, UGT1A3-catalyzed chenodeoxycholic acid 24-acyl-glucuronidation, and UGT2B7-catalyzed naloxone 3-glucuronidation with IC_50_ values of 3.9, 4.0, 4.3, and 10.0 µM, respectively, and showed mechanism-based inhibition of CYP2C8-catalyzed amodiaquine *N*-deethylation with a *K_i_* value of 2.1 µM. It negligibly inhibited CYP1A2, CYP2A6, CYP2B6, CYP2C19, CYP2D6, UGT1A1, UGT1A4, UGT1A6, and UGT1A9 activities at 50 μM in human liver microsomes. These in vitro results indicate that AM-2201 needs to be examined for potential pharmacokinetic drug interactions in vivo due to its potent inhibition of CYP2C8, CYP2C9, CYP3A4, UGT1A3, and UGT2B7 enzyme activities.

## 1. Introduction

**Figure 1:**
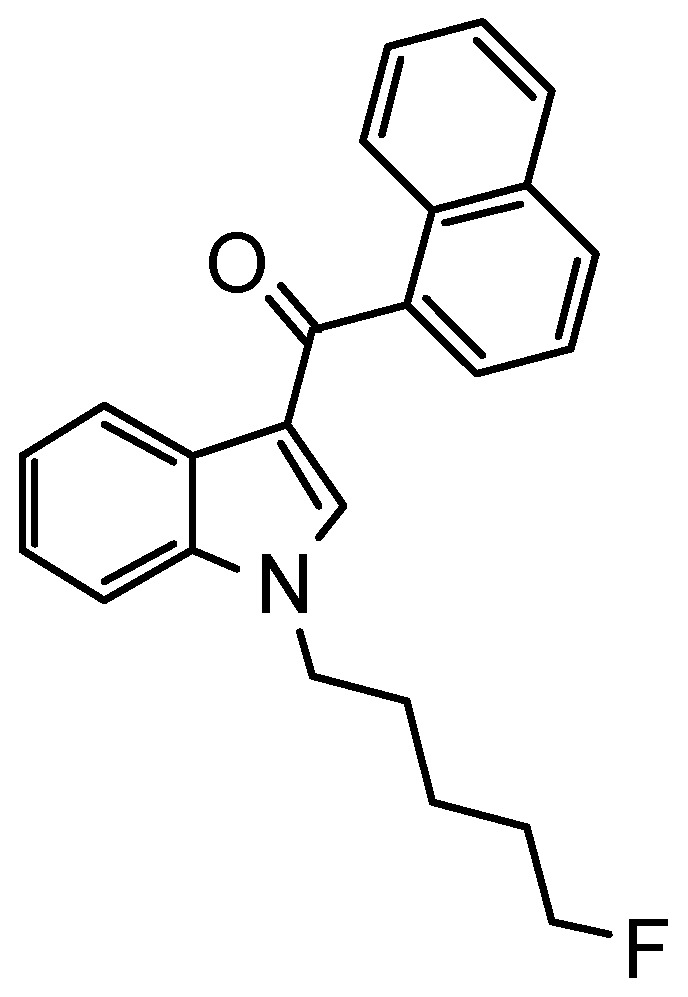
Chemical structure of AM-2201.

Synthetic cannabinoids are a group of substances with functionally similar effects to Δ9-tetrahydrocannabinol (THC), which is responsible for the major psychoactive effects of cannabis, and generally bind to cannabinoid receptor type 1 (CB_1_) or 2 (CB_2_) [1]. The synthetic cannabinoid JWH-018 was first detected in herbal smoking mixtures, called Spice, in 2008; 160 synthetic cannabinoids are now monitored by the European Monitoring Centre for Drugs and Drug Addiction (EMCDDA) through the EU Early Warning System [2]. The continued emergence of synthetic cannabinoids on the recreational and illicit drug markets has caused unexpected and serious events and has become a global public health issue [3,4,5,6,7,8,9]. AM-2201 (Figure 1) is a third-generation synthetic cannabinoid, modified by introduction of a fluorine atom to JWH compounds, and exerts potent pharmacological actions on brain function, causing psychoactive and intoxicating effects [10].

It has been increasingly found in recreational users and intoxication cases, as well as herbal products marketed for recreational use, such as incense blends [3,6,7,11,12,13,14,15,16,17,18]. Cytochrome P450 (CYP) 1A2 and CYP2C9 enzymes play major roles in the metabolism of AM-2201 to 4-hydroxyfluoropentyl-AM-2201, AM-2201 pentanoic acid, and 5-hydroxypentyl-AM-2201 [19].

Variability of drug metabolism due to inhibition and induction of uridine 5'-diphospho-glucuronosyltransferase (UGT) enzymes, as well as CYP enzymes, is an important complicating factor in pharmacology and toxicology, drug therapy, environmental exposure, and risk assessment [20,21]. Phytocannabinoids, such as THC, cannabidiol, and cannabinol, inhibit CYPs 1A1, 1A2, 2A6, 2B6, 2C9, 2D6, 3A4, and 3A5 activities in human liver microsomes and recombinant CYP enzymes; cannabidiol is the most potent inhibitor of many CYPs [22,23,24,25,26,27,28,29]. Cannabidiol inhibited UGT1A9 and UGT2B7 activities, and cannabinol inhibited UGT1A9 activity in human liver and intestine microsomes and recombinant UGT enzymes [30]. Understanding the roles of synthetic cannabinoids in the regulation of CYP and UGT is necessary to predict individual differences in synthetic cannabinoid toxicity and to prevent toxic drug–drug interactions; however, the effects of synthetic cannabinoids, including AM-2201, on the regulation of CYP and UGT enzymes remain largely unknown.

In this study, the inhibitory effects of AM-2201 on eight major human CYP activities (CYPs 1A2, 2A6, 2B6, 2C8, 2C9, 2C19, 2D6, and 3A4) and six major UGT activities (UGTs 1A1, 1A3, 1A4, 1A6, 1A9, and 2B7) were examined using pooled human liver microsomes to evaluate the possibility of AM-2201-induced drug interactions.

## 2. Results and Discussion

AM-2201 potently inhibited CYP2C9-catalyzed diclofenac 4′-hydroxylation and CYP3A4-mediated midazolam 1′-hydroxylation, with IC_50_ values of 11.9 and 6.9 µM, respectively, and moderately inhibited CYP2C8-catalyzed amodiaquine *N*-deethylation with an IC_50_ value of 53.8 µM in human liver microsomes (Table 1).

**Figure 2:**
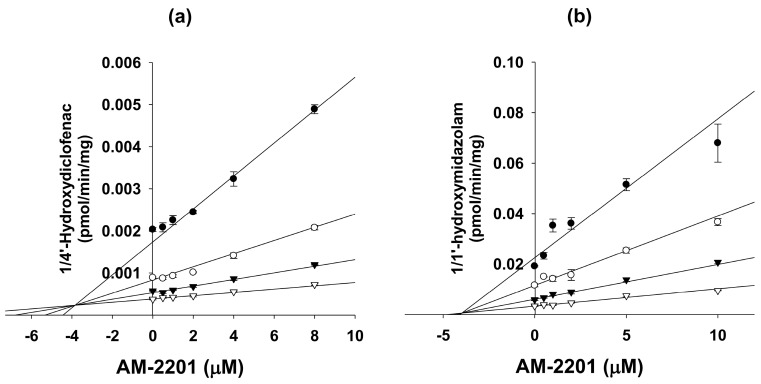
Representative Dixon plots for the inhibitory effect of AM-2201 on (**a**) CYP2C9-catalyzed diclofenac 4′-hydroxylation and (**b**) CYP3A4-catalyzed midazolam 1′-hydroxylation activities in ultrapool human liver microsomes. Each symbol represents a substrate concentration: (**a**) diclofenac: ●, 2 μM; ◯, 5 μM; ▼, 10 μM; ▽, 20 μM and (**b**) midazolam: ●, 1 μM; ◯, 2 μM; ▼, 4 μM; ▽, 8 μM. Data represent the mean ± standard deviation (SD) (*n* = 3).

AM-2201 negligibly inhibited CYP1A2-mediated phenacetin *O*-deethylation, CYP2A6-mediated coumarin 7-hydroxylation, CYP2B6-mediated bupropion hydroxylation, CYP2C19-mediated [*S*]-mephenytoin 4′-hydroxylation, and CYP2D6-mediated bufuralol 1′-hydroxylation activities at 50 µM in human liver microsomes (Table 1). AM-2201 competitively inhibited CYP2C9-catalyzed diclofenac 4′-hydroxylation and CYP3A4-catalyzed midazolam 1′-hydroxylation, with *K*_i_ values of 3.9 and 4.0 μM, respectively (Figure 2, Table 1).

**Figure 3:**
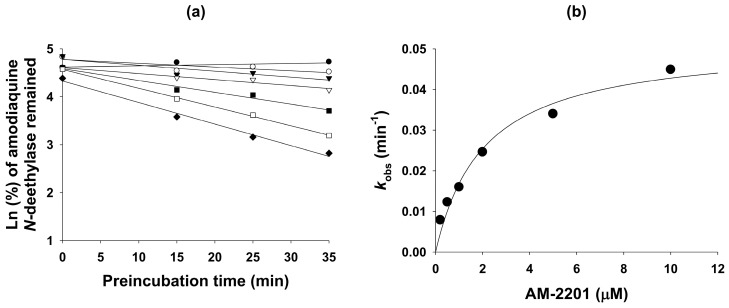
(**a**) Inactivation kinetics of the formation of *N*-deethylamodiaquine from amodiaquine in human liver microsomes by the following AM-2201 concentrations: ●, 0 μM; ◯, 0.2 μM; ▼, 0.5 μM; ▽, 1 μM; ■, 2 μM; ☐, 5 μM; ♦, 10 μM and (**b**) the relationship between *k*_obs_ and AM-2201 concentrations to estimate *k*_inact_ and *K*_i_ values of CYP2C8-mediated amodiaquine *N*-deethylation. Data were derived from two replicates.

AM-2201 lowered the IC_50_ value of CYP2C8-catalyzed amodiaquine *N*-deethylation more than 2.5-fold after a 30-min pre-incubation with human liver microsomes and NADPH, compared with values obtained without pre-incubation (Table 1), indicating that AM-2201 acted as a potent mechanism-based inhibitor of CYP2C8. AM-2201 decreased CYP2C8-mediated amodiaquine *N*-deethylation with increasing pre-incubation time in a concentration-dependent manner (Figure 3) with *k*_inact_ and apparent *K*_i_ values of 0.052 min^−1^ and 2.1 µM, respectively (Table 1).

**Figure 4:**
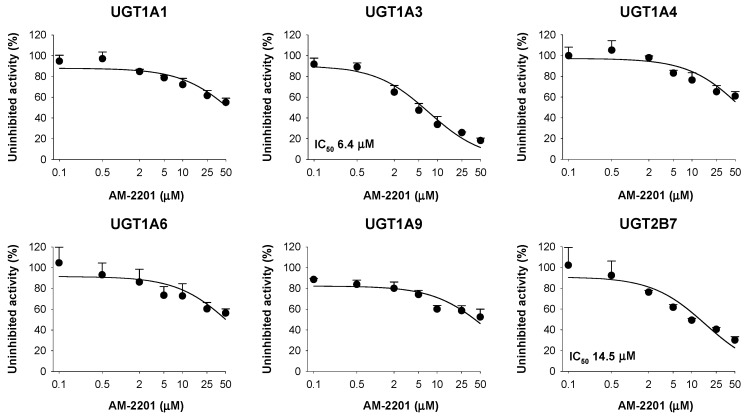
Inhibitory effect of AM-2201 on six UGT metabolic activities in ultrapool human liver microsomes with IC_50_ values. Cocktail substrate concentrations used for the assessment of IC_50_ were as follows: 0.5 μM SN-38 for UGT1A1, 2 μM chenodeoxycholic acid for UGT1A3, 0.5 μM trifluoperazine for UGT1A4, 1 μM *N*-acetylserotonin for UGT1A6, 0.2 μM mycophenolic acid for UGT1A9, and 1 μM naloxone for UGT2B7. Data represent the mean ± SD (*n* = 3).

The inhibitory effects of AM-2201 on six major human UGT enzymes were evaluated using human liver microsomes (Figure 4). AM-2201 potently inhibited UGT1A3-catalyzed chenodeoxycholic acid 24-acyl-glucuronidation and UGT2B7-catalyzed naloxone 3-β-d-glucuronidation in human liver microsomes, with IC_50_ values of 6.4 and 14.5 µM, respectively (Figure 4).

**Figure 5:**
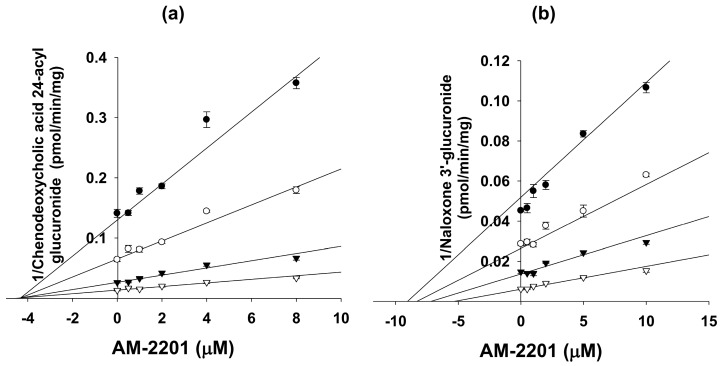
Representative Dixon plots for the inhibitory effects of AM-2201 on (**a**) UGT1A3-catalyzed chenodeoxycholic acid 24-acyl glucuronidation and (**b**) UGT2B7-catalyzed naloxone 3′-glucuronidation in human liver microsomes. Each symbol represents the substrate concentration: (**a**) chenodeoxycholic acid, ●, 1 μM, ◯, 2μM, ▼, 5 μM, ▽, 10 μM; and (**b**) naloxone; ●, 0.5 μM; ◯, 1 μM; ▼, 2 μM; ▽, 5 μM. Data represent the mean ± SD (*n* = 3).

AM-2201 negligibly inhibited UGT1A1-catalyzed SN-38 glucuronidation, UGT1A4-catalyzed trifluoperazine *N*-glucuronidation, UGT1A6-catalyzed *N*-acetylserotonin glucuronidation, and UGT1A9-catalyzed mycophenolic acid glucuronidation at 50 µM. AM-2201 competitively inhibited UGT1A3-catalyzed chenodeoxycholic acid 24-acyl-glucuronidation, with a *K*_i_ value of 4.3 μM, and showed mixed inhibition of UGT2B7-catalyzed naloxone 3-β-d-glucuronidation, with a *K*_i_ value of 10.0 μM, in human liver microsomes (Figure 5).

This in vitro study is the first to show the inhibitory potential of the popularly-used synthetic cannabinoid AM-2201 on CYP and UGT enzymes in human liver microsomes. AM-2201 was a potent competitive inhibitor of CYP2C9-catalyzed diclofenac hydroxylation with *K*_i_ value of 3.9 µM in human liver microsomes (Table 1, Figure 2). *K*_i_ values for the inhibition of THC, cannabinol, and cannabidiol on CYP2C9-catalyzed diclofenac hydroxylation in human liver microsomes were 1.31 µM, 1.29 µM, and 9.88 µM, respectively [27]. The *K*_i_ value (4.0 µM) for the competitive inhibition of AM-2201 on CYP3A4-catalyzed midazolam 1′-hydroxylation in human liver microsomes was comparable to the *K*_i_ (6.14 µM) of cannabidiol for CYP3A4-catalyzed diltiazem *N*-demethylation, but the IC_50_ values of THC and cannabinol for CYP3A4 activity were more than 50 µM [24].

Based on these in vitro results, AM-2201 may cause drug interactions with CYP2C9 substrates such as celecoxib, diclofenac, glyburide, losartan, tolbutamide, torasemide, and *S*-warfarin [31], and CYP3A4 substrates including atorvastatin, cyclosporine, clarithromycin, estradiol, felodipine, lovastatin, nifedipine, simvastatin, and tacrolimus [32].

AM-2201 was a potent mechanism-based inhibitor of CYP2C8, with a *K*_i_ value of 2.1 µM and its inhibitory potency was comparable to that of selective CYP2C8 inhibitor, quercetin (*K*_i_, 2.0 µM) [33], but was less than those produced by phenelzine (*K*_i_, 54.3 µM) [34] and gemfibrozil glucuronide (*K*_i_, 20–52 µM) [35]. These results indicate that AM-2201 may inhibit the metabolism of drugs metabolized by CYP2C8, such as cerivastatin, paclitaxel, repaglinide, and sorafenib [36]. However, the inhibitory effects of THC, cannabinol, and cannabidiol on CYP2C8 activity were not evaluated, to our knowledge.

AM-2201 negligibly inhibited CYP1A2, CYP2A6, CYP2B6, CYP2C19, and CYP2D6 activities at 50 µM in human liver microsomes. However, phytocannabinoids such as THC, cannabidiol, and cannabinol showed potent inhibition of CYP2B6 activity with *K*_i_ values of 2.81 µM, 0.694 µM, and 2.55 µM, respectively [25], and cannabidiol competitively inhibited CYP2D6 activity with a *K*_i_ value of 2.42 µM in human liver microsomes [26].

AM-2201 showed the potent competitive inhibition (*K*_i_, 4.3 µM) of UGT1A3-catalyzed chenodeoxycholic acid 24-acyl glucuronidation similar to a selective UGT1A3 inhibitor, glycyrrhetinic acid (IC_50_, 4.3 μM) in human liver microsomes, indicating that this compound should be used carefully with UGT1A3 substrates, such as chenodeoxycholic acid, fimasartan, losartan, candesartan, zolarsartan, and JWH-018 [37,38,39,40], to avoid possible drug interactions. 

The inhibitory potency (*K*_i_, 10.0 µM) of AM-2201 on UGT2B7-catalyzed naloxone 3′-glucuronidation was similar to that (*K*_i,_ 9.8 μM) of cannabidiol for UGT2B7-catalyzed ethanol glucuronidation [30], suggesting that AM-2201 may cause drug interactions with UGT2B7 substrates, such as morphine, zidovudine, efavirenz, ethanol, carbinol, JWH-018, and flurbiprofen [30,40,41,42,43,44].

There is no reported systemic information on human AM-2201 pharmacokinetics, essential for the prediction of AM-2201-induced drug interaction potential, but plasma and blood concentrations for AM-2201 were reported to be 0.14 nM to 26.4 nM in recreational users and intoxication cases [7,45]. However, plasma or blood concentrations do not reflect tissue concentrations, particularly liver concentrations. As AM-2201 is extensively metabolized [19], its metabolites may inhibit CYP and UGT activities. Consequently, these in vitro results suggest that AM-2201 should be examined in terms of potential in vivo pharmacokinetic drug interactions caused by inhibition of CYP2C8, CYP2C9, CYP3A4, UGT1A3, and UGT2B7 activities.

## 3. Materials and Methods

### 3.1. Materials and Reagents

AM-2201 was purchased from Cayman Chemical Company (Ann Arbor, MI, USA). Acetaminophen, alamethicin, chenodeoxycholic acid, coumarin, diclofenac, 7-hydroxycoumarin, midazolam, mycophenolic acid, *N*-acetylserotonin, naloxone, naloxone 3-β-d-glucuronide, NADPH, phenacetin, trifluoperazine, Trizma^®^ base, and uridine 5′-diphospho-glucuronic acid (UDPGA) were purchased from Sigma-Aldrich (St. Louis, MO, USA). Ultrapool human liver microsomes (150 donors), ^13^C_2_,^15^N-acetaminophen, bufuralol, *N*-desethylamodiaquine, 1′-hydroxybufuralol, 4-hydroxy-diclofenac, 4-hydroxymephenytoin, d_3_-4-hydroxymephenytoin, 1′-hydroxymidazolam, d_9_-1-hydroxybufuralol, and [*S*]-mephenytoin were obtained from Corning Life Sciences (Woburn, MA, USA). SN-38 was obtained from Santa Cruz Biotechnology (Dallas, TX, USA). *N*-Acetylserotonin β-d-glucuronide, chenodeoxycholic acid-24-acyl-β-glucuronide, mycophenolic acid β-d-glucuronide, and SN-38 glucuronide were obtained from Toronto Research Chemicals (Toronto, ON, Canada). Acetonitrile and methanol (high performance liquid chromatography (HPLC) grade) were obtained from Fisher Scientific (Fair Lawn, NJ, USA). All other chemicals were of the highest quality available.

### 3.2. Inhibitory Effect of AM-2201 on Eight Major CYP Activities in Human Liver Microsomes

The inhibitory potencies (IC_50_ values) of AM-2201 on CYP1A2, CYP2A6, CYP2C8, CYP2C9, CYP2C19, CYP2D6, and CYP3A4 activities were evaluated in pooled human liver microsomes using a cocktail of seven CYP substrates and liquid chromatography-tandem mass spectrometry (LC-MS/MS). The incubation mixtures were prepared in total volumes of 100 µL as follows: pooled human liver microsomes (0.2 mg/mL), 1.0 mM NADPH, 10 mM MgCl_2_, 50 mM potassium phosphate buffer (pH 7.4), various concentrations of AM-2201 in dimethyl sulfoxide (DMSO; final concentrations of 0.1–50 µM, DMSO <1% *v*/*v*), and a cocktail of seven CYP probe substrates, as described previously [46]. The CYP substrates were used at concentrations approximating their respective *K*_m_ values: 50 µM phenacetin, 2.5 µM coumarin, 2.0 µM amodiaquine, 10 µM diclofenac, 100 µM (*S*)-mephenytoin, 5 µM bufuralol, and 2.5 µM midazolam. After a 3-min pre-incubation at 37 °C, the reactions were initiated by addition of NADPH and incubation proceeded for 15 min at 37 °C in a shaking water bath. The reaction was stopped by placing the tubes on ice and adding 100-µL amounts of ice-cold methanol containing internal standards (^13^C_2_,^15^N-acetaminophen for acetaminophen and *N*-desethylamodiaquine, d_9_-1′-hydroxybufuralol for 4′-hydroxydiclofenac, and 7-hydroxycoumarin, 4′-hydroxy-mephenytoin, 1′-hydroxybufuralol, and 1′-hydroxymidazolam). The incubation mixtures were centrifuged (13,000× *g*, 4 min, 4 °C). All assays were performed in triplicate and mean values were used in calculations.

To measure the mechanism-based inhibition of CYP activities, various concentrations of AM-2201 (0.1–50 µM) were pre-incubated for 30 min with human liver microsomes in the presence of NADPH. Each reaction was initiated by adding the seven-CYP probe substrate cocktail.

The inhibitory effects (IC_50_ values) of AM-2201 on CYP2B6-catalyzed bupropion 4-hydroxylase activity were determined in ultrapool human liver microsomes using LC-MS/MS [46]. The incubation mixtures were prepared in a total volume of 100 μL as follows: pooled human liver microsomes (0.2 mg/mL), 1.0 mM NADPH, 10 mM MgCl_2_, 50 mM potassium phosphate buffer (pH 7.4), various concentrations of AM-2201 (final concentrations of 0.1–50 μM, acetonitrile concentration <1% *v*/*v*), and the CYP2B6-selective substrate bupropion (50 μM). After a 3-min pre-incubation at 37 °C, the reactions were initiated by adding a NADPH generating system and incubated for 15 min at 37 °C in a shaking water bath. The reaction was stopped by placement of the tubes on ice and addition of 100 μL of ice-cold methanol containing internal standards (d_9_-1-hydroxybufuralol for 4-hydroxy-bupropion). The incubation mixtures were then centrifuged (13,000× *g*, 4 min, 4 °C). All incubations were performed in triplicate, and average values were used.

To evaluate mechanism-based inhibition of CYP2B6 activity, various concentrations of AM-2201 (final concentrations of 0.1–50 μM, acetonitrile concentration <1% *v*/*v*) were pre-incubated for 30 min with human liver microsomes in the presence of NADPH. The reaction was started by addition of the CYP2B6 probe substrate, bupropion.

### 3.3. Inhibitory Effects of AM-2201 on Six Major UGT Activities in Human Liver Microsomes

The inhibitory effects of AM-2201 on UGT1A1, UGT1A3, UGT1A4, UGT1A6, UGT1A9, and UGT2B7 activities were evaluated by LC-MS/MS using incubation with a cocktail of UGT substrates in ultrapool human liver microsomes, as described previously [47]. Each incubation mixture was prepared in a final volume of 100 µL as follows: pooled human liver microsomes (0.2 mg/mL), 5 mM UDPGA, 10 mM MgCl_2_, 50 mM Tris buffer (pH 7.4), various concentrations of AM-2201 in acetonitrile (final concentrations of 1–50 µM, acetonitrile <1% *v*/*v*), and a UGT enzyme-specific substrate from a cocktail set (A set: 0.5 µM SN-38, 2 µM chenodeoxycholic acid, and 0.5 µM trifluoperazine; B set: 1 µM *N*-acetylserotonin, 0.2 µM mycophenolic acid, and 1 µM naloxone). After 3 min of pre-incubation at 37 °C, the reactions were initiated by addition of UDPGA; incubation continued for 60 min at 37 °C in a shaking water bath. The reaction was stopped by placing the tubes on ice and adding 50 µL ice-cold acetonitrile containing internal standards (propofol glucuronide for chenodeoxycholic acid 24-acyl-β-glucuronide and mycophenolic acid glucuronide, and meloxicam for SN-38 glucuronide, trifluoperazine glucuronide, *N*-acetylserotonin β-d-glucuronide, and naloxone 3-β-d-glucuronide). The incubation mixtures were centrifuged (13,000× *g*, 4 min, 4 °C). All assays were performed in triplicate and average values were used in calculations.

### 3.4. Mechanism-based Inhibition of CYP2C8 Activity by AM-2201

The mechanism-based inhibitory effect of AM-2201 on CYP2C8 activity was further evaluated using time- and concentration-dependent inhibition assays in human liver microsomes. The microsomes (1 mg/mL) were pre-incubated with various concentrations of AM-2201 in 50 mM potassium phosphate buffer (pH 7.4) in the presence of NADPH and aliquots (10 μL) of the pre-incubated mixtures were withdrawn at 15, 25, and 35 min after incubation commenced and added to other tubes containing 2 µM amodiaquine, 1 mM NADPH, 50 mM potassium phosphate buffer (pH 7.4), and 10 mM MgCl_2_ in 90 μL reaction mixtures. The second reaction was terminated after incubation for 10 min by adding 100-µL amounts of ice-cold methanol containing d_9_-1′-hydroxybufuralol. The incubation mixtures were centrifuged (13,000× *g*, 4 min, 4 °C), and then 50 µL of each supernatant was diluted with 50 µL of water. Aliquots (5 µL) of the diluted supernatants were analyzed by LC-MS/MS.

### 3.5. Kinetic Analysis

To determine *K*_i_ values of AM-2201 for CYP2C9 and CYP3A4, human liver microsomes (0.15 mg/mL) were incubated with various concentrations of substrates (2–20 μM diclofenac for CYP2C9 and 1–8 μM midazolam for CYP3A4), 1 mM NADPH, 10 mM MgCl_2_, and various concentrations of AM-2201 in 50 mM potassium phosphate buffer (pH 7.4) in a total incubation volume of 100 μL. The reactions were initiated by addition of NADPH at 37 °C and stopped after 10 min by placing the incubation tubes on ice and adding 100 μL of ice-cold methanol containing an internal standard (d_9_-1-hydroxybufuralol). The incubation mixtures were centrifuged (13,000× *g*, 4 min, 4 °C) and then 50 μL of the supernatant was diluted with 50 μL of water. Aliquots (5 μL) of the diluted supernatants were analyzed by LC-MS/MS.

To determine *K*_i_ values of AM-2201 for UGT1A3 and UGT2B7 enzymes, human liver microsomes (0.15 mg/mL) were incubated with various concentrations of chenodeoxycholic acid (1–10 μM) for UGT1A3 or of naloxone (0.5–5 μM) for UGT2B7, 5 mM UDPGA, 10 mM MgCl_2_, and various concentrations of AM-2201 in 50 mM Tris buffer (pH 7.4) in a total incubation volume of 100 μL. The reactions were initiated by addition of UDPGA at 37 °C and stopped after 60 min by placing the incubation tubes on ice and adding 100 μL of ice-cold methanol containing propofol glucuronide (internal standard for UGT1A3) or meloxicam (internal standard for UGT2B7). The incubation mixtures were centrifuged (13,000× *g*, 4 min, 4 °C), and then, 50 μL of the supernatant was diluted with 50 μL of water. Aliquots (5 μL) of the diluted supernatants were analyzed by LC-MS/MS.

### 3.6. LC-MS/MS Analysis

A tandem mass spectrometer (TSQ Quantum Access; Thermo Scientific, San Jose, CA, USA) equipped with an electrospray ionization (ESI) source, coupled to a Nanospace SI-2 LC system (Tokyo, Japan), was used. The column and autosampler temperatures were 50 °C and 6 °C, respectively.

The metabolites formed from CYP substrates were quantified simultaneously by a LC-MS/MS method described previously [46]. The ESI source settings in positive-ion mode for metabolite ionization were: capillary voltage, 4200 V; vaporizer temperature, 350 °C; capillary temperature, 330 °C; sheath gas pressure, 35 psi; and auxiliary gas pressure, 15 psi. Quantification was performed by selected reaction monitoring (SRM) of the [M + H]^+^ ion and the related product ion for each metabolite: acetaminophen, 152.1 > 110.3; *N*-desethylamodiaquine, 328.1 > 283.0; 7-hydroxycoumarin, 163.0 > 107.2; 4-hydroxybupropion, 256.1 > 238.0; 4′-hydroxydiclofenac, 312.0 > 231.1; 4′-hydroxy-mephenytoin; 235.1 > 150.1; 1′-hydroxybufuralol, 278.1 > 186.1; 1′-hydroxymidazolam, 341.9 > 324.0; ^13^C_2_,^15^N-acetaminophen 155.1 > 111.2; and d_9_-1′-hydroxybufuralol, 287.2 > 187.0. Analytical data were processed using Xcalibur software (version 2.1, Thermo Scientific).

The metabolites formed from the six UGT cocktail substrates were measured simultaneously using the LC-MS/MS method [47]. The ESI source settings in both positive- and negative-ion modes for metabolite ionization were: capillary voltage, 4200 V; vaporizer temperature, 350 °C; capillary temperature, 330 °C; sheath gas pressure, 35 psi; and auxiliary gas pressure, 15 psi. Each metabolite was quantified via SRM in the negative-ion mode: chenodeoxycholic acid 24-acyl-β-glucuronide, 567.2 > 391.0, mycophenolic acid glucuronide, 495.2 > 318.9, propofol glucuronide (IS), 353.3 > 177.1; and positive ion-mode: SN-38 glucuronide, 569.0 > 393.0, trifluoperazine glucuronide, 584.2 > 408.1, *N*-acetylserotonin-β-d-glucuronide, 395.2 > 219.0, naloxone 3-β-d-glucuronide, 504.0 > 310.0, meloxicam (IS), 352.0 > 115.1. Data were processed using Xcalibur software.

### 3.7. Data Analysis

IC_50_ values (concentration of inhibitor causing 50% inhibition of the original enzyme activity) were calculated using the SigmaPlot program (ver. 11.0; Systat Software, Inc., San Jose, CA, USA). The apparent kinetic parameters for inhibitory potential (*K*_i_ and *k*_inact_ values) and inhibition mode were estimated from the fitted curves using the Enzyme Kinetics program (ver. 1.1; Systat Software Inc., San Jose, CA, USA).

## 4. Conclusions

AM-2201 potently inhibited CYP2C9-catalyzed 4′-hydroxylation, CYP3A4-catalyzed midazolam 1′-hydroxylation, UGT1A3-catalyzed chenodeoxycholic acid 24-acyl-glucuronidation, and UGT2B7-catalyzed naloxone 3-glucuronidation, with *K_i_* values of 3.9, 4.0, 4.3, and 10.0 µM, respectively, and showed potent mechanism-based inhibition of CYP2C8-catalyzed amodiaquine *N*-deethylation, with a *K_i_* value of 2.1 µM. AM-2201 should be examined in terms of potential in vivo pharmacokinetic drug interactions attributable to its inhibition of CYP2C8, CYP2C9, CYP3A4, UGT1A3, and UGT2B7 activities.

## Figures and Tables

**Table 1:** Inhibitory effect of AM-2201 on major CYP metabolic activities in human liver microsomes.

Marker Enzymes	CYP	IC_50_ (μM)		*K_i_* (μM) (*K*_inact_, min^−1^ or Inhibition Mode)
		No Preincubation	With Preincubation *	
Phenacetin *O*-deethylase	1A2	NI	NI	-
Coumarin 7-hydroxylase	2A6	NI	NI	-
Bupropion hydroxylase	2B6	NI	21.9	-
Amodiaquine *N*-deethylase	2C8	53.8	6.9	2.1 (*k*_inact_: 0.0516)
Diclofenac 4′-hydroxylase	2C9	11.9	11.9	3.9 (competitive)
[*S*]-Mephenytoin 4′-hydroxylase	2C19	NI	31.3	-
Bufuralol 1′-hydroxylase	2D6	NI	NI	-
Midazolam 1′-hydroxylase	3A4	6.9	3.8	4.0 (competitive)

* AM-2201 was preincubated for 30 min in the presence of reduced β-nicotinamide adenine dinucleotide phosphate (NADPH) before the addition of the substrate. NI: no inhibition, inhibition <50% at 50 μM of AM-2201. Cocktail substrate concentrations used for the assessment of IC_50_ were as follows: 50 μM phenacetin, 2.5 μM coumarin, 2.5 μM amodiaquine, 10 μM diclofenac, 100 μM [*S*]-mephenytoin, 5.0 μM bufuralol, and 2.5 μM midazolam. The inhibition of CYP2B6 activity was evaluated separately using 50 μM bupropion. Data were derived from the average of three determinations.
